# The Impact of Extracellular Ca^2+^ and Nanosecond Electric Pulses on Sensitive and Drug-Resistant Human Breast and Colon Cancer Cells

**DOI:** 10.3390/cancers13133216

**Published:** 2021-06-28

**Authors:** Julita Kulbacka, Nina Rembiałkowska, Anna Szewczyk, Helena Moreira, Anna Szyjka, Irutė Girkontaitė, Kamil P. Grela, Vitalij Novickij

**Affiliations:** 1 Department of Molecular and Cellular Biology, Faculty of Pharmacy, Wroclaw Medical University, 50-367 Wroclaw, Poland; nina.rembialkowska@umed.wroc.pl (N.R.); a.szewczyk@umed.wroc.pl (A.S.); 2 Department of Animal Developmental Biology, Institute of Experimental Biology, University of Wroclaw, 50-137 Wroclaw, Poland; 3 Department of Basic Medical Science, Faculty of Pharmacy, Wroclaw Medical University, 50-367 Wroclaw, Poland; helena.moreira@umed.wroc.pl (H.M.); anna.szyjka@umed.wroc.pl (A.S.); 4 Department of Immunology, State Research Institute Centre for Innovative Medicine, 08406 Vilnius, Lithuania; irute.girkontaite@imcentras.lt; 5 Department of Drugs Form Technology, Faculty of Pharmacy, Wroclaw Medical University, 50-367 Wroclaw, Poland; kamil.grela@umed.wroc.pl; 6 Institute of High Magnetic Fields, Vilnius Gediminas Technical University, 10223 Vilnius, Lithuania

**Keywords:** calcium ions, electroporation drug resistance, human adenocarcinoma, membrane permeabilization

## Abstract

**Simple Summary:**

The drug resistance phenomenon in cancer constantly induces problems in therapeutic protocols. Pulsed electric fields (PEFs) seem to be a promising method in drug molecule delivery. Here we have proved that electroporation supported by calcium ions can alternate the activity of drug resistance proteins. Our results indicated that MDR1 expression is not significantly modified by nanosecond electroporation in multidrug-resistant cells. However, PEF significantly inhibited MDR1 activity and cell viability when combined with calcium ions.

**Abstract:**

(1) Background: Calcium electroporation (CaEP) is based on the application of electrical pulses to permeabilize cells (electroporation) and allow cytotoxic doses of calcium to enter the cell. (2) Methods: In this work, we have used doxorubicin-resistant (DX) and non-resistant models of human breast cancer (MCF-7/DX, MCF-7/WT) and colon cancer cells (LoVo, LoVo/DX), and investigated the susceptibility of the cells to extracellular Ca^2+^ and electric fields in the 20 ns–900 ns pulse duration range. (3) Results: We have observed that colon cancer cells were less susceptible to PEF than breast cancer cells. An extracellular Ca^2+^ (2 mM) with PEF was more disruptive for DX-resistant cells. The expression of glycoprotein P (MDR1, P-gp) as a drug resistance marker was detected by the immunofluorescent (CLSM) method and rhodamine-123 efflux as an MDR1 activity. MDR1 expression was not significantly modified by nanosecond electroporation in multidrug-resistant cells, but a combination with calcium ions significantly inhibited MDR1 activity and cell viability. (4) Conclusions: We believe that PEF with calcium ions can reduce drug resistance by inhibiting drug efflux activity. This phenomenon of MDR mechanism disruption seems promising in anticancer protocols.

## 1. Introduction

Multidrug resistance (MDR) of cancer cells is a high-priority biomedical problem that is constantly being discussed [1]. MDR is the most significant factor in a successful chemotherapeutic anticancer treatment protocol; however, it is not a straightforward problem to overcome this. The available data indicate that throughout chemotherapeutic clinical procedures, a significant reduction of the cytotoxic effect of a variety of chemotherapeutic agents can be observed, including anthracyclines, vinca alkaloids, and epipodophyllotoxins [2,3]. This phenomenon is called acquired drug resistance and commonly regards breast cancer [4]. The second type of resistance-primary or intrinsic resistance is caused by the primary overexpression of specific proteins and protein pumps that effectively remove drug molecules from the cells [5]. In this group, we can include colon and rectal cancer [6]. In general, this phenomenon we can call “multidrug drug resistance”. The cells’ ability to become resistant to a variety of cytotoxic drugs is frequently a consequence of a lower drug concentration inside a cell due to an increased drug efflux [7]. Drug-resistant cells often present a higher expression of molecular “pumps” on their membranes which can pump out drugs used in chemotherapy [8]. As a result, various types of cancer depending on MDR require a specific treatment strategy, such as surgery, radiotherapy, or chemotherapy. Currently, several defined mechanisms implicate this phenomenon, e.g., the family of ABC transporters [9], mutations, enhanced DNA repair systems, gene amplification, epigenetic altering, or miRNAs in gene dysregulation. However, the most commonly known and verified are the ABC transporters, particularly P-glycoprotein (P-gp, MDR1) [10]. The problem is severe because more than 50 mammalian ABC transporters having ATP-binding cassettes exist. These proteins can occur in normal cells too. Moreover, cancer cells show resistance to different drugs even before treatment, referred to as intrinsic multidrug resistance, which is typical in colon cancer [11]. Lastly, cells can acquire resistance to drugs as a consequence of the long-term therapy [12].

The available literature shows that physical methods, e.g., ultrasonic techniques (sonoporation) [13,14] or electroporation [15,16,17] in combination with chemotherapy, or electromagnetic treatment against antibiotic [18] or antimicrobial resistance [19], can be efficiently applied to overcome cell resistance in vivo and in vitro. Electrochemotherapy is based on the application of high-voltage electric pulses, causing permeabilization of membranes (electroporation) and increased drug uptake by cancer cells [20]. It is a pulse-dependent phenomenon, and usually, the microsecond range pulses are used [21]. However, in recent years, the interest in nanosecond range procedures has been increasing due to better pulse energy control, primarily non-thermal treatment, reduced muscle contractions, and improved electric-field distribution homogeneity [22,23]. The research of chemotherapeutic compounds which have synergy with electroporation is also constantly performed, and the interest in calcium as an antitumor chemotherapeutic agent has increased [24]. It was shown that calcium ions, when used in high concentrations (>100 mM), can be successfully applied in electrochemotherapy for the treatment of various types of tumors [25,26]. Nevertheless, the MDR phenomenon during electrochemotherapy is not widely covered, while different types of cancer cells have different susceptibility to the treatment [27,28].

Our study hypothesized that electroporation in the nanosecond range modulates the functioning of drug resistance mechanisms at the membrane level, and the use of electroporation with Ca^2+^ ions can be more impairing for drug-resistant cells. We have also investigated the feasibility of nanosecond pulses and compared them to the microsecond range procedure. We have also investigated the feasibility of nanosecond pulses and compared them to the microsecond range procedure. The treatment’s efficiency was judged by evaluation of cell membrane permeabilization rate, viability, immunofluorescent staining, and transport activity of P-glycoprotein.

## 2. Materials and Methods

### 2.1. Pulsed Power Setup and PEFs Protocols

**Figure 1:**
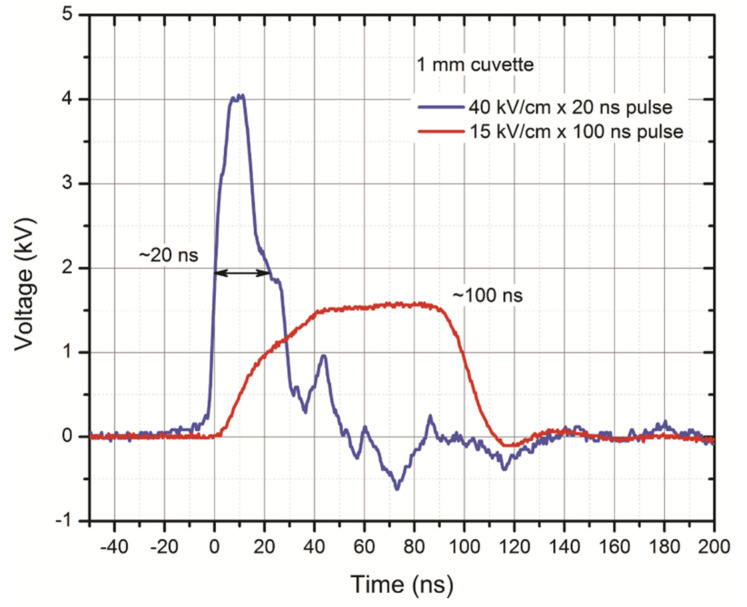
Waveforms of the pulses applied in the study; **red** color represents signal of 100 ns pulse (15 kV/cm) and **navy blue** corresponds to 20 ns pulse (40 kV/cm).

The experimental setup consisting of a 3 kV, 100 ns–1 ms square-wave high-voltage pulse generator (VGTU, Vilnius, Lithuania) and a commercially available electroporation cuvette with a 1 mm gap between electrodes (Biorad, Hercules, USA) was used. For a 20 ns pulse delivery, the PPG-20 generator (FID Technology, Germany) was applied. The voltage that was applied to the cuvette was varied in the 0.14–6 kV range, corresponding to a 1.4–60 kV/cm electric field. The pulses were delivered in bursts of 200 at 1 kHz for the 100–400 ns, 5–10 kV/cm protocols and in bursts of 200–1200 at 0.2 kHz frequency for 40/60 kV/cm × 20 ns protocols. For the final experiments, the following protocols were used: EP1 –10 kV/cm × 300 ns × 200; EP2–40 kV/cm × 20 ns × 400; EP3–60 kV/cm × 20 ns × 400. The 1.2 kV/cm × 100 μs × 8 microsecond pulses were used as a reference (EP4). EP4 protocol corresponds to the ESOPE standard applied in clinical practice [29]. The waveforms to highlight the rise and fall times of the pulses are shown in Figure 1. As can be seen, the 20 ns pulse features a quick transient process during the fall time which is due to a slight load impedance mismatch.

### 2.2. Cell Culture

Two human counterpart cell lines were selected for the experiment: breast and colon cancer. The studies were performed in vitro on a doxorubicin-sensitive (MCF-7/WT) and doxorubicin-resistant type (MCF-7/DX) of human breast adenocarcinoma cell line obtained from the Department of Tumor Biology, Comprehensive Cancer Center, Maria Sklodowska-Curie Memorial Institute in Gliwice (Poland), and a doxorubicin-sensitive (LoVo) and doxorubicin-resistant (LoVo/DX) type of human colon adenocarcinoma obtained from Ludwik Hirszfeld Institute of Immunology and Experimental Therapy, Polish Academy of Sciences (Wroclaw, Poland). MCF-7/WT and MCF-7/DX cells were grown in DMEM (Sigma, Poland), supplemented with 10% fetal bovine serum (Lonza BioWhittaker, Switzerland) and penicillin/streptomycin (Sigma, Poland). LoVo/DX and MCF-7/DX were obtained from parental counterparts by exposure to increasing concentrations of DOX according to the protocol [30]. LoVo and LoVo/DX cells were maintained in Ham’s F-12 (Sigma, Poland) supplemented with 10% fetal bovine serum (FBS, Lonza BioWhittaker, Switzerland) and 1% penicillin/streptomycin (Sigma, Poland). Cell cultures were cultured as a monolayer on a 25 and 75 cm^2^ plastic flask (Sarstedt, Germany), maintained in a humidified atmosphere at 37 °C and 5% CO_2_, and detached for the experiments by trypsinization (trypsin 0.025% and EDTA 0.02% solution, Sigma, Poland). Cells were passed every 2–3 days and a day before the experiment.

### 2.3. The Evaluation of Calcium and Magnesium Ion Content in Experimental Buffers

The assay of Ca^2+^ and Mg^2+^ in every buffer solution was evaluated via complexometric titration with standardized Na_2_-EDTA solution in a pH-controlled environment [31,32,33]. Here we determined the buffer solutions used in the electroporation experiments, i.e.,: Milli-Q class deionized water (control); 10 mM HEPES, 10 mM HEPES with sucrose and 1 mM Mg^2+^; and 10 mM HEPES with sucrose, 1 mM Mg^2+^, and 2 mM CaCl_2_. At first, the sum of dissociated Ca^2+^ and Mg^2+^ ions was measured at pH 10 against eriochrome black T (solochrome black) as an indicator. A total color change from wine red to blue or greenish-blue (since it was the final color being inflected by the buffer composition) was considered as a titration end point. Secondly, the assay of Ca^2+^ alone was evaluated from a separate aliquot, at pH 12, against murexide as an indicator. A total color change from pink to violet was considered as a titration end point. The assay of Mg^2+^ alone was calculated as a difference between the result of the first titration (Ca^2+^ + Mg^2+^) and second titration (Ca^2+^ only). Every titration was performed in triplicate using an over-titrated sample as an end point color reference. The “blank” titration was also performed in each of the above methods and the final results were corrected upon it. Every solution or dilution was performed using Milli-Q class deionized water which was free from measurable traces of Ca^2+^ or Mg^2+^. The pH was controlled by addition of concentrated ammonium chloride buffer (final pH 10) or 1 mol/L NaOH solution (final pH 12–13). The measurements are presented in Table 1.

### 2.4. Cell Permeabilization Rate Evaluation Using Flow Cytometry

Flow cytometry analysis was performed to evaluate electroporation efficacy by assessing of the ability of cells to internalize impermeant dye-YO-PRO-1. Before the application of electric pulses, the cells were incubated on ice for 20 min. In the context of in vitro electroporation, keeping the cells on ice before application of the pulses is beneficial since it allows lower cell metabolism and thus the pH changes in the stock sample can be neglected. Before electroporation, YO-PRO™-1 iodide (YP-1, λ_exc_491/λ_em_509, Thermo Scientific, Poland) was added to the cell suspension. The concentration of YP-1 was 1 μM, which was prepared in the SKM buffer of low conductivity (0.12 S/m) (pH 7.4; 10 mM phosphate KH_2_PO_4_/K_2_HPO_4_ (POCH, Gliwice, Poland), 1 mM magnesium chloride (MgCl_2_, cat. no.: Sigma M8266), 250 mM sucrose (C_12_H_22_O_11_, Chempur, Poland)). After pulsing, the cells were incubated at room temperature for 10 min, followed by flow cytometry or fluorescence microscopy analysis. Flow cytometric analysis was performed using a CyFlow CUBE-6 flow cytometer (Sysmex, Poland). The samples were excited using the 488-nm line of the blue laser and the fluorescence of YP-1 was measured with an FL-1 detector. The control samples without treatment were used as a negative control for gate definition. After permeabilization, depending on the applied protocol, a fluorescent spectrum shift due to dye uptake was observed. Data were analyzed using CyView software (Sysmex). All experiments were repeated at least three times.

### 2.5. The Effect of PEF and Extracellular Ca^2+^

For electroporation, the cells were trypsinized and centrifuged (5 min, 1000 rpm, MPW-341 Centrifuge with a stable rotor, MPW Med. Instruments, Poland). For each sample, 5 × 10^5^ of cells were resuspended in HEPES buffer (10 mM HEPES (C_8_H_18_N_2_O_4_S, Sigma-Aldrich, cat. no.: H337), 250 mM sucrose (C_12_H_22_O_11_, Chempur, Poland), and 1 mM magnesium chloride (MgCl_2_, Sigma, M8266) in sterile MilliQ water. The manufacturer of the sucrose indicated cation traces where calcium content may not exceed 0.002% of Ca^2+^ (0.05 mM). This value was considered as a control level. Cell suspension was kept on ice before electroporation and then pulses were delivered in a cuvette (Bio-Rad) between 1 mm gap parallel plate aluminum electrodes. Electroporation protocols were also combined with CaCl_2_ (Sigma-Aldrich) in a 2 mM concentration, based on our previous study [34]. Calcium chloride solution was prepared in the same buffer as for electroporation alone. After pulsing, 10 min incubation at 37 °C was performed. Then the cells were resuspended in the appropriate cell culture medium (DMEM or Ham’s F12) for further evaluation. Untreated controls were handled in the same way as treated cells, all steps were performed in the same time, and the same culture dishes were used (cuvettes, centrifugation tubes). For the MTT assay (Section 2.6), cells were seeded into 96-well microculture plates (density: 4 × 10^4^ of cells in 200 µL of culture medium/well) (Nunc, Denmark). For the immunocytochemical assay (Section 2.7), cells were seeded into 10-well microscopic slides as described in the protocols below.

### 2.6. Viability Assay

The MTT assay was performed 24 or 72 h post electroporation to determine cell viability of breast or colon cancer cells as a viability marker. First, the cells were incubated with 100 μL of the MTT [3-(4,5-Dimethylthiazol-2-yl) -2,5-Diphenyltetrazolium Bromide] reagent (Sigma, Poland) at 37 °C for 1.5 h. Then, formazan crystals were dissolved with the addition of 100 μL of acidic isopropanol and mixed. The absorbance was measured at 570 nm using a multi-well plate reader (GloMax^®^ Discover, Promega, Madison, WI, USA). The results were presented as a percentage compared to the untreated control cells. Experiments were repeated three times in triplicate.

### 2.7. P-glycoprotein and Cell Membrane Fluorescence Imaging

The confocal laser scanning microscope (CLSM) was used to semi-quantitatively evaluate P-glycoprotein (P-gp, MDR1) as the main marker of drug resistance promotion by immunofluorescence studies. Cells after treatment with EP alone or combined with calcium were resuspended on microscopic cover slides in 35 mm Petri dishes and incubated for 24 h to adhere. Then, cells were rinsed with PBS, fixed in 4% formalin (10 min), washed 3× by PBS, and permeabilized with 0.5% Triton X-100 in PBS for 5 min. After, cells were washed with PBS 3 × 5 min and blocked with 1% bovine serum albumin (BSA, Sigma-Aldrich, Poland, Poznan) in PBS for 1 h. The following antibodies were used: primary antibody monoclonal anti-MDR1 (1:200; Santa Cruz, USA) for overnight incubation at 4 °C, and secondary antibody Fluorescein (FITC)-conjugated AffiniPure Fragment Donkey Anti-Mouse IgG (cat. no.: 715-095-150), where the incubation was for 60 min, at RT (1:100; Jackson ImmunoResearch Laboratories, Biokom, Poland, Wroclaw). Then, cells were washed 2× with PBS and then incubated for 15 min with CellMask Deep Red (1:1000; Thermo Fisher, C10046) to visualize the distribution and structure of the membranes. The cells were mounted in a fluorescence mounting medium (Fluoroshield™, Sigma Aldrich, F6182). For the imaging, a confocal laser scanning microscope (Olympus FluoView FV1000) was used. MDR1 (FITC) was detected using 473 nm excitation wavelength and 520 nm emission wavelength, and a deep red membrane marker was detected by 635 nm excitation wavelength and 693 nm emission wavelength. All experiments were performed in four independent repetitions. Fiji package of ImageJ 1.52 p software (ROI Manager, Multi Measure) [35] was used for the quantification of the mean fluorescent signal. This method is based on the signal intensity analysis and from each cell, divided by the counted object number. A minimum of 10^2^ cells were analyzed from each slide. Before the analysis, the background intensity was removed to avoid signal interference.

### 2.8. Rhodamine 123 Accumulation Studies—A Marker of P-gp Activity

Intracellular content of rhodamine 123 (Sigma-Aldrich, St. Louis, MO, USA) was evaluated by flow cytometry, as previously reported [36,37]. The working rhodamine 123 solution was freshly prepared prior to each experiment by dissolving 1 mg in distilled water and then diluting in a complete culture medium to the final concentration of 5 µM. Briefly, the treated and control cells were removed from the culture flask using TrypleTM Express solution (GIBCO, Waltham, MA, USA), spun down, and pelleted. The cells were then resuspended in 1 mL of rhodamine 123 solution in plastic Falcon tubes. The samples were incubated for 1 h at 37 °C in a CO_2_-incubator. Following the incubation time, the cells were washed once with ice-cold HBSS (4 °C) (Hank’s Balanced Salt Solution, Lonza, Basel, Switzerland) and then resuspended in 0.5 mL of ice-cold HBSS. The samples were immediately analyzed with a CyFlow^®^ SPACE flow cytometer (Sysmex, Kobe, Prefektura Hyōgo, Japan) using 488 nm (50 mW) laser excitation and a 536/40 (BP) filter for rhodamine fluorescence detection. The results were analyzed using FCS express 4 flow software (De Novo Software, Glendale, CA, USA). Results were expressed as a mean fluorescence intensity (MIF) and normalized to the control (E0) according to previous study [37]. Experiments were repeated a minimum of three times.

### 2.9. Statistical Analysis

In all experiments, one-way analysis of variance (ANOVA) was used to compare different treatments. For the analysis of the fluorescent MDR1 staining, results were compared to the control untreated cells, expressing the basic level of this protein. Tukey HSD and Sidak’s multiple comparison tests for the evaluation of the differences was used when ANOVA indicated a statistically significant result (*p* < 0.05 or *p* < 0.005 depending on the experiment were considered as statistically significant. The data was post-processed in OriginPro software (OriginLab, Northampton, MA, USA) and GraphPad Prism 7.0 software (GraphPad Software, San Diego, CA, USA). All experiments were performed at least in triplicate and the treatment results were expressed as mean ± standard deviation.

## 3. Results

The purpose of this study was to investigate the dependence of cell permeabilization on the applied electric field parameters and the alternation on the drug resistance phenomenon in the used cell models after the PEF exposure.

### 3.1. Cells’ Susceptibility to Membrane Electropermeabilization

**Figure 2:**
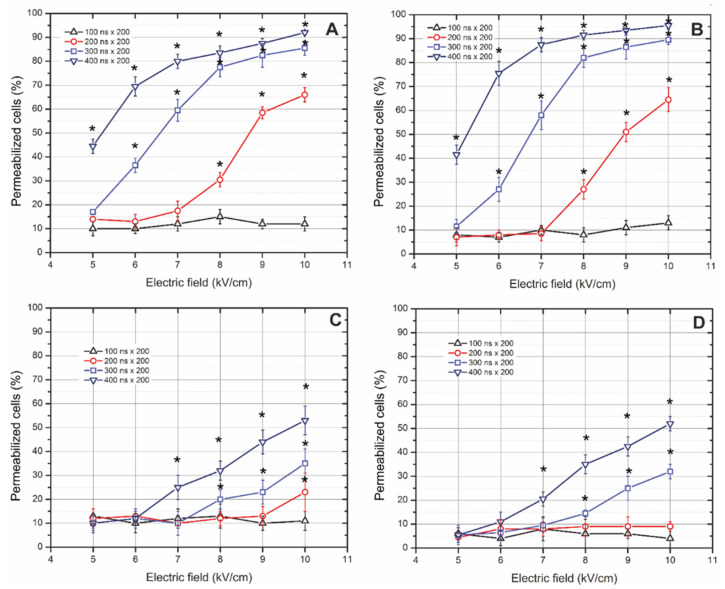
The fraction of YO-PRO-1 permeable cells 10 min post electric field treatment evaluated using flow cytometry, where (**A**) doxorubicin-sensitive (MCF-7/WT) and (**B**) doxorubicin-resistant (MCF-7/DX) human breast adenocarcinoma cells; (**C**) doxorubicin-sensitive (LoVo) and (**D**) doxorubicin-resistant (LoVo/DX) human colon cancer cells. Asterisk (*) corresponds to statistically significant (*p* < 0.05) difference versus untreated control. The percentage of permeabilized cells shows the fraction of permeabilized cells compared to all the cells in each sample.

The pulses were supplied in bursts and the uptake of YO-PRO-1 was evaluated. The results for all four cell lines are summarized in Figure 2. As it can be seen in Figure 2A,B, the susceptibility of doxorubicin-resistant breast adenocarcinoma cells and non-resistant ones to pulsed electric field varies only slightly. In most of the cases, the response is similar (*p* > 0.05). However, the most notable difference was observed between breast and colon cancer cells (Figure 2A,B vs. Figure 2C,D). It can be seen that 100 ns pulses are not effective for permeabilization of these cells in the 5–10 kV/cm range, while a further increase of pulse duration and total burst energy showed a dose-dependent pattern. The 300 ns pulses allowed to cover the whole range of permeabilization efficacies and precisely control the electric field amplitude’s permeabilization rate. For example, the 5 kV/cm × 300 ns × 200 pulses protocol resulted in <20% of cells being permeable, while the 10 kV/cm protocol triggered more than 85% permeabilization. The equivalent pulse parameters were acquired for 400 ns bursts, i.e., the 85% permeabilization can be reached already at 8 kV/cm. Similarly, the response of human colon carcinoma (LoVo and LoVo/DX) was investigated (Figure 2C,D). As can be seen, the cells are much more treatment-resistive compared to breast adenocarcinoma cell lines. However, the responses between doxorubicin-resistive and sensitive cells are identical, highlighting the advantages of physical methods. Nevertheless, even the highest nanosecond treatment intensity (applied in this study) resulted only in half of the cells being permeable to YO-PRO-1.

**Figure 3:**
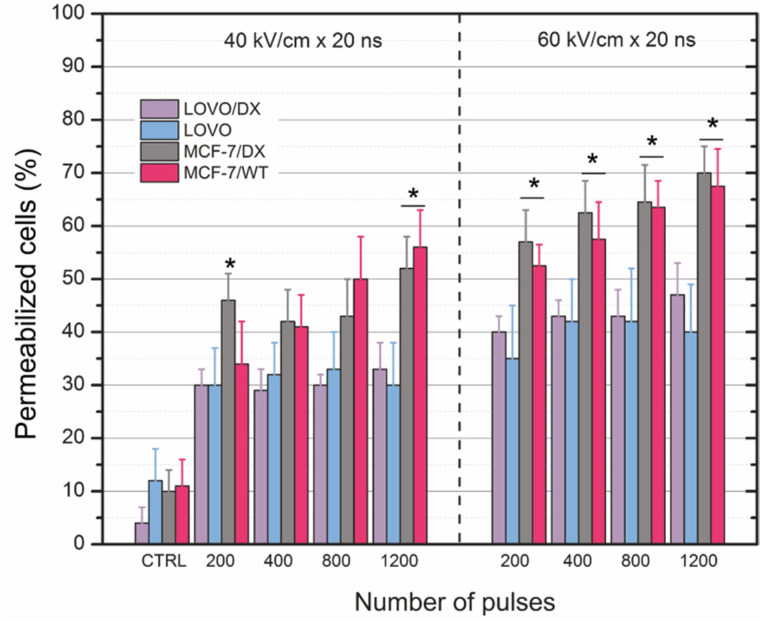
The fraction of YO-PRO-1 permeable cells 10 min post nanosecond-electric field treatment evaluated using flow cytometry in MCF-7/WT, MCF-7/DX, LoVo, and LoVo/DX cells. Asterisk (*) corresponds to statistically significant (*p* < 0.05) difference between two types of cells (MCF and LoVo). The percentage of permeabilized cells shows the fraction of permeabilized cells compared to the whole cells’ portion in each sample.

The short nanosecond protocols (20 ns) also showed a definitive dose response in terms of permeabilization (Figure 3). As was shown in Figure 3, increasing the electric field’s amplitude from 40 to 60 kV/cm results in a 10–20% higher permeabilization rate. Likewise, to sub-microsecond protocols, there were no statistically significant differences between doxorubicin-resistant and non-resistant cell lines. We could not observe an absolute dependence of treatment efficiency on the number of pulses. With an increase of the PEF amplitude, the fluorescence intensity of the cells also increased.

### 3.2. PEF Effects with/without Calcium Ions on Cell Viability

**Figure 4:**
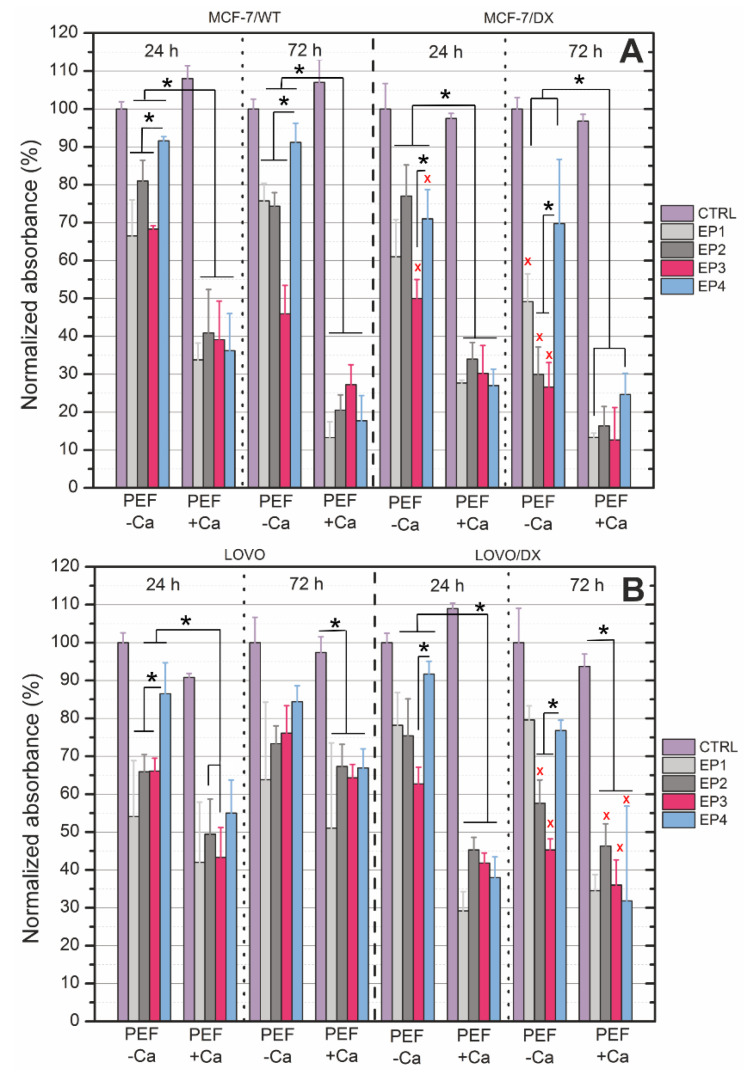
The viability of cells (**A**) MCF-7/WT and MCF-7/DX; (**B**) LoVo and LoVo/DX exposed to electroporation and calcium ions measured my MTT assay depending on the treatment protocol, where EP1–10 kV/cm × 300 ns × 200; EP2–40 kV/cm × 20 ns × 400; EP360 kV/cm × 20 ns × 400; EP4–1.2 kV/cm × 100 µs ns × 8 (ESOPE); CTRL–untreated control. All the data is normalized to untreated control. Asterisk (*) corresponds to statistically significant difference (*p* < 0.05) between protocols (EP1–EP4) with and without calcium; (x) corresponds to statistically significant difference (*p* < 0.05) between non-resistant cells and DX-resistant cells.

Furthermore, the study was limited to four protocols, EP1–EP4 (EP1–10 kV/cm × 300 ns × 200; EP2–40 kV/cm × 20 ns × 400; EP3–60 kV/cm × 20 ns × 400; EP4–1.2 kV/cm × 100 µs ns × 8), and the cells were electroporated with 2 mM CaCl_2_. We performed the titration of the buffers used in electroporation to determine calcium and magnesium ion content. The obtained results are shown in Table 1. It was noted that pure 10 mM HEPES was contaminated by trace ions. However, the manufacturer of sucrose indicated that this reagent can contain no more than 0.002% of calcium ions. It was verified, and in HEPES-based buffer, 0.005 mM of calcium ion concentration was detected. In our experimental study, we did not exceed 2 mM calcium concentration. The cell viability was evaluated 24 and 72 h post-treatment. Results for electroporation with calcium ions are presented for the breast adenocarcinoma in Figure 4A and colon carcinoma in Figure 4B. As shown in Figure 4, for all the cell lines, electroporation induced higher viability decrease, particularly when combined with calcium ions. The inactivation rate was higher after 72 h in most of the cases.

What is important is that nanosecond pulses alone caused a significant decrease in cell proliferation after 72 h. ESOPE (European Standard Operating Procedures on Electrochemotherapy) protocol (EP4) also reduced cell viability but less intensively than nsPEF, while EP1 (10 kV/cm × 300 ns × 200) had the strongest impact in most of the treated samples. The presence of external calcium ions provoked a strong viability decrease, up to 4-fold in the case of EP1 (72 h) in breast cancer cells and 1.5-fold in colon cancer cells (72 h). Resistant cell lines (derived from colon and breast) were slightly more sensitive. Therefore, PEF + Ca^2+^ reduced cell viability 3.5-fold in MCF-7/DX and 2.3-fold in LoVo/DX cells. The tendency of PEF protocols (EP1-EP4) when combined with calcium ions for the corresponding pairs of cells (MCF-7/WT vs. MCF-7/DX and LoVo vs. LoVo/DX) were comparable (±10%). The response of both MCF-7 cell lines was comparable, independently of DOX resistance. The exposure to PEF with calcium decreased wild-type cells’ viability to ca. 40% after 24 h and ca. 20% after 72 h. In MCF-7/DX cells, there was a noted decrease to ca. 30% after 24 h and to ca. 15–20% after 72 h. Thus, resistant cells were only slightly more sensitive to Ca-PEF. LoVo cell lines were more resistive to the treatment as compared to the MCF-7. PEF with calcium ions reduced LoVo cells’ viability to 50% after 24 h and 60–70% after 72 h. In resistant cells, the viability was lowered to ca. 40% after 24 h and 72 h. Thus, the most evident difference was observed in LoVo vs. LoVo/DX cells after 72 h (Figure 4B).

### 3.3. The Effect of PEF Combined with Calcium Ions on P-glycoprotein

**Figure 5:**
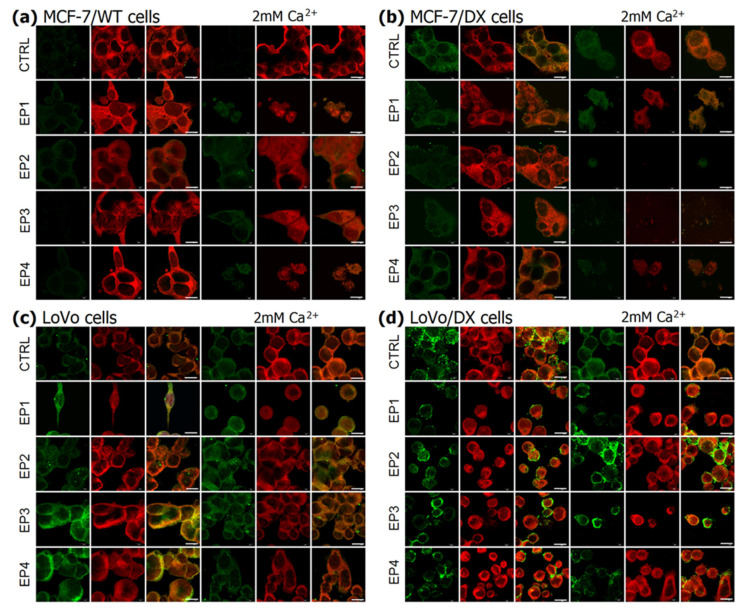
Immunofluorescent analysis of MDR1 protein double-stained with CellMask membrane marker in human breast adenocarcinoma cells, (**a**) MCF-7/WT; (**b**) MCF-7/DX and human colon carcinoma cells; (**c**) LoVo; (**d**) LoVo/DX. The following parameters were applied without/with 2 mM Ca^2+^: EP1–10 kV/cm × 300 ns × 200; EP2–40 kV/cm × 20 ns × 400; EP3–60 kV/cm × 20 ns × 400; EP4–1.2 kV/cm × 100 µs ns × 8 (ESOPE). **Red** fluorescence corresponds to the DeepRed^®^CellMask cell membrane marker, **green** fluorescence corresponds to MDR1 protein.White scale bar corresponds to 10 µm.

The immunofluorescent analysis was employed in this study. The verification of the resistance phenomena was based on the immunofluorescent assay of P-glycoprotein and then visualized by confocal microscopy. The red cell membrane marker (CellMask Deep Red) was used to verify colocalization of green fluorescence emitted from P-gp (membrane protein) and to verify if MDR1 distribution could be affected (increased, decreased, or rearranged) by PEF with or without calcium ions. This double staining is crucial in varying resistance levels and MDR1 expression in sensitive (MCF-7/WT and LoVo) and resistant cells (MCF-7/DX and LoVo/DX). Although the red membrane marker fluorescence is not reduced in electroporated cells, green MDR1 fluorescence significantly changes. The results for breast cancer cells are presented in Figure 5a,b, and the results for colon cancer cells are shown in Figure 5c,d. All controls are shown in the upper panel (CTRL). In both resistant cell types (breast and colon), an increased P-glycoprotein expression was observed in untreated controls. Sensitive MCF-7/WT cells did not exhibit a fluorescent signal and LoVo cells exhibited only a trace signal. MCF-7/WT cells after electroporation with calcium ions showed a slight fluorescence signal. In sensitive colon cancer cells (LoVo), an increase in MDR1 signal was observed after electroporation alone and a slight decrease after adding extracellular calcium ions. In resistant cells, the initial level of MDR1 protein was detected, and after electroporation, expressly with calcium ions, the signal was weakening, e.g., after EP2, EP3, and EP4 in MCF-7/DX and after EP1, EP3, and EP4 in LoVo/DX.

**Figure 6:**
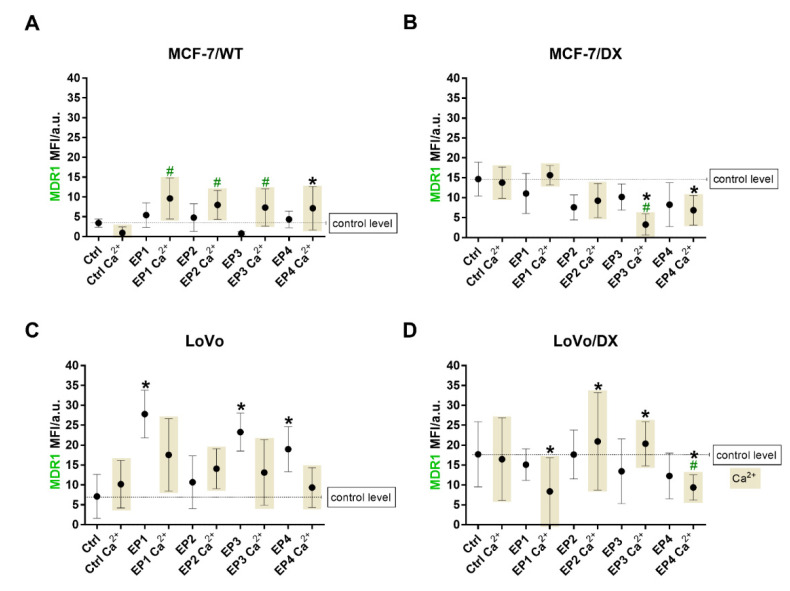
The analysis of fluorescent P-gp (MDR1) signal from breast cancer cells (**A**) MCF-7/WT; (**B**) MCF-7/DX, and colon cancer cells (**C**) LoVo and (**D**) LoVo/DX. The following parameters were applied without/with 2 mM Ca^2+^: EP1–10 kV/cm × 300 ns × 200; EP2–40 kV/cm × 20 ns × 400; EP3–60 kV/cm × 20 ns × 400; EP4–1.2 kV/cm × 100 µs ns × 8 (ESOPE). Dotted line represents the control level. Signal was examined by ImageJ software. Grey fields indicate samples with calcium ions. Asterisk (* and **#**) corresponds to statistically significant (*p* < 0.05) difference. (*) in relation to the untreated control cells; (**#**) in relation to calcium-treated control.

The mean fluorescent signal intensity (MFI) corresponding to mean MDR1 amount was presented in Figure 6. Only green fluorescence (MDR1) was analyzed, as this is the only variable. ImageJ analysis indicated the most significant decrease in MCF-7/DX after EP3 and EP4 with calcium ions. The MDR1 signal derived from LoVo and LoVo/DX cells indicated the most significant decrease after the exposure of EP1 and EP4 with calcium ions.

**Figure 7:**
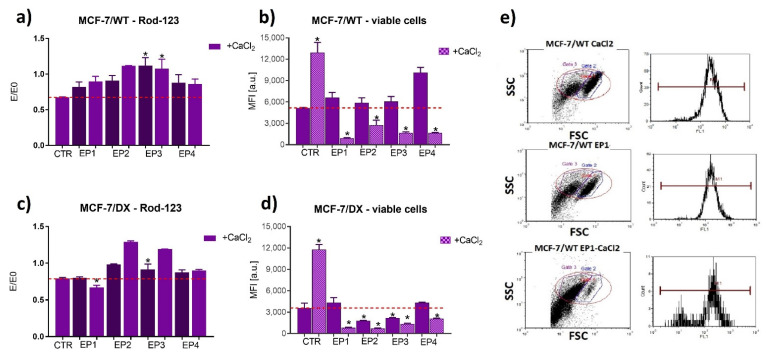
Rhodamine 123 accumulation assay and viability in breast adenocarcinoma (**a**,**b**) sensitive and (**c**,**d**) resistant cells after 24 h post electroporation with/without CaCl_2_. Results were normalized to control untreated cells (E/E0 where E0 = Ctrl, E–(MFI) mean fluorescence intensity of the appropriate sample). Exemplary dot plots and histograms are shown in (**e**), where X axis corresponds to FSC (forward scatter-diameter of the cells), and Y axis to SSC (side scatter-internal complexity of the cells. The following parameters were applied: EP1–10 kV/cm × 300 ns × 200; EP2–40 kV/cm × 20 ns × 400; EP3–60 kV/cm × 20 ns × 400; EP4–1.2 kV/cm × 100 µs ns × 8 (ESOPE), * p ≤ 0.05. E/E0 (Rod-123 uptake) and MFI results are taken from Gate 2 (blue); Gate 1 indicates dead or damaged cells; Gate 3 (purple) separates the analyzed cells from the debris.

**Figure 8:**
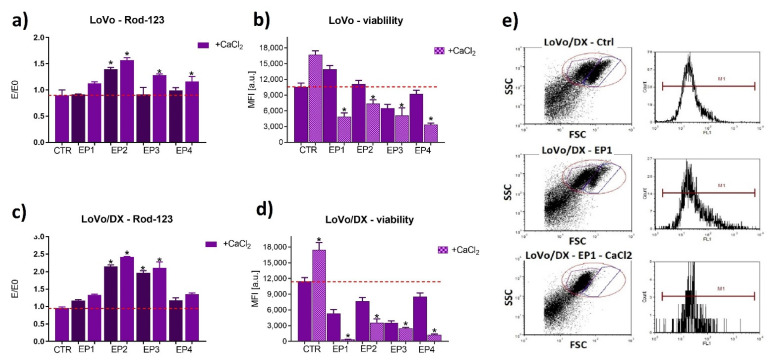
Rhodamine 123 accumulation assay and viability in colon carcinoma (**a**,**b**) sensitive and (**c**,**d**) resistant cells after 24 h post electroporation with/without CaCl_2_. Results were normalized to control untreated cells (E/E0 where E0 = Ctrl, E–(MFI) mean fluorescence intensity of the appropriate sample). Exemplary dot plots and histograms are shown in (**e**), where X axis corresponds to FSC (forward scatter-diameter of the cells), and Y axis to SSC (side scatter-internal complexity of the cells. The following parameters were applied: EP1–10 kV/cm × 300 ns × 200; EP2–40 kV/cm × 20 ns × 400; EP3–60 kV/cm × 20 ns × 400; EP4–1.2 kV/cm × 100 µs ns × 8 (ESOPE), * p ≤ 0.05. E/E0 (Rod-123 uptake) and MFI results are taken from Gate 2 (blue); Gate 1 indicates dead or damaged cells; Gate 3 (purple) separates the analyzed cells from the debris.

To verify the activity of P-gp, the efflux assay with rhodamine 123 was used. This method is based on cytofluorimetric measurements and enables simultaneous validation of cell condition and viability (FSC and SSC parameters). Figure 7 and Figure 8 represent results from the rhodamine 123 assay performed by flow cytometry. In our study, a Rod-123 efflux study corresponds to P-gp activity as a “drug pump”. This assay was applied to determine the transport function of P-glycoprotein. The values E/E0 normalized to the control (E0) represent Rod-124 accumulation—an indicator of P-gp activity. The higher the accumulation, the lower the activity of the drug resistance pump. Rhodamine 123 is a substrate of P-gp. Recent reports indicate that rhodamine 123 (Rod-123) may also be a substrate of other multidrug resistance-associated proteins [37,38,39,40].

The obtained results indicated that individual electroporation parameters inhibited P-gp transport function, which was revealed by an increased accumulation of Rod123 in the case of all parameters for MCF-7/WT and MCF-7/DX cells, EP2 (20 ns, 40 kV/cm, 400 pulses) for LoVo and LoVo/DX, and EP3 (20 ns, 60 kV/cm, 400 pulses) for LoVo/DX. The effect was stronger in the case of electroporation with calcium ions and in the case of resistant cells. The experiment performed by flow cytometry also enabled us to evaluate cell viability 24 h after electroporation (Figure 7b,d and Figure 8b,d) (SSC to FSC graph, exemplary marked red area on dot-plots Figure 7e and Figure 8e). The exposure to calcium ions alone stimulated viability in all four cell lines (Figure 7b,d and Figure 8b,d) compared to non-treated cells. The viability dropped significantly after electroporation with calcium chloride, in particular in DOX-resistant cell lines.

## 4. Discussion

This study shows that electroporation in combination with calcium ions can alternate drug resistance in cancer cells. The anticancer effect of pulsed electric fields with calcium ions was previously demonstrated in various cancers [25,41,42], but not in drug resistance cells. It was demonstrated that the nanosecond range pulses could be used for effective cell-membrane permeabilization for sensitive cells and cells with drug resistance. Interestingly, our results revealed that a high electric field (40 and 60 kV/cm) with 20 ns pulses could diminish P-glycoprotein activity. This effect was not associated with the immunofluorescent reactivity of MDR1 protein. Thus, we suppose that nsPEF could inhibit or modulate the efficacy of drug pumps related to ABC transporters (ATP-binding cassette (ABC) transporter superfamily including P-gp, MRP1, and BCRP) [43,44]. Chemotherapeutic drugs can relatively easily diffuse across the cell membrane based on passive diffusion. ABC-transporters then act as efflux pumps in the membranes of cancer cells, and their overexpression and activity are responsible for the reduced drug uptake [45]. Selective inhibitors or modulators (e.g., verapamil, elacridar) can be applied to reverse these pumps’ activity [46]. Unlike chemical factors, electroporation supported by calcium ions seems a reasonable method to control and overcome drug resistance.

Our study confirmed that cell membranes in various cancer cell lines differ in sensitivity to nsPEF. The level of membrane permeabilization was significantly higher in breast cancer cells than in colon cancer cells which is attributed rather to the morphological difference between the cells, i.e., cell size, cell membrane composition, and fluidity [47], but not the drug resistance. The available experimental and theoretical data also confirm that larger cells are more willingly permeabilized [48,49]. According to the viability results, we could indicate that EP1 (300 ns, 10 kV) and EP3 (20 ns, 60 kV) revealed the strongest anticancer effect. Electroporation with calcium ions (2 mM CaCl_2_) significantly intensified the difference in survival rate between cell lines but the difference between separate protocols (EP1-4) was not high (±10%). However, the electrophoretic component of longer (microsecond) pulses is much higher compared to ultra-short pulses; therefore, a similar treatment outcome indicates that passive diffusion of calcium was the dominant mechanism of delivery through a permeabilized membrane. Indeed, calcium is a small ion, and thus passive diffusion is sufficient to ensure significant electrotransfer, even in the nanosecond range when the electrophoresis influence is negligible [34]. We have shown that during supra-electroporation, when the pulse duration is significantly shorter than the polarization time, electric field strength should be increased several-fold to trigger the same permeabilization rates as in the case of longer pulses. It is an expected result, while the capability to derive equivalent pulse parameters for any pulse duration opens opportunities for development of new (shorter-pulse) protocols which will ensure less muscle contractions, bioimpedance mitigation, and thus more uniform exposure for the tumors.

Our previous study has shown that colon cancer with doxorubicin resistance can be efficiently exposed to microsecond electroporation to support the anticancer DOX effect. We have also demonstrated that the electroporation technique induced changes in P-gp expression [50]. In the case of human breast cancer with the same type of resistance, we used microsecond electroporation to enhance photodynamic reaction efficacy with NIR-cyanines. It was shown that MCF-7/DX cells were more susceptible to electroporation than wild-type breast cancer cells. Similar to our research, increased MDR1, GSTpi, and MRP7 protein levels after electroporation of breast cancer cells were reported [51]. The authors indicated that one of the factors that differ between normal and cancer cells is the fraction and localization of the negatively charged phospholipid PS [52].

Up to now, the activity of a drug resistance protein after nanosecond electroporation was not reported. Based on the P-gp activity (Rod-123) we can state that PEF can block the function of this drug transporter. Here we show that nsPEF can be used as a temporary MDR controlling tool to obtain better drug uptake, and calcium supports this effect. Possibly, the membrane-stabilizing effects of calcium [53,54] also impact drug resistance proteins. Levine et al. demonstrated that calcium ions induced conformational changes of PS in mixed bilayers [53]. We suppose that this phenomenon will not be inert to the membrane proteins, including the ABC-transporter superfamily. The group of Pakhomova et al. revealed that necrosis was dependent on the increased calcium content (2 mM) and the osmotically-independent pore expansion. It was also stated that even new or larger pores could be formed without membrane destruction [55]. Thus, this pore expansion seems favorable in the case of resistant cells. Cemazar et al. performed studies on radioresistant tumors in vivo and obtained a good response using ESOPE protocol with cisplatin or bleomycin [44,45,46]. Similar research was carried out by Condello et al. on almost the same cell model, including a mitomycin-resistant cell line. The authors show morphological alternations in cells exposed to microsecond electroporation by AFM visualization [56,57]. It was also indicated that nanosecond pulses caused recruitment of intracellular Ca^2+^ [58]. It was previously observed that mitochondria and other membranous cell organelles are “porated” during exposure to nanosecond pulses [22,59,60,61]. The authors also observed that after applying nsPEF, calcium ions are released from internal calcium-stores such as ER and mitochondria or taken up from outside the cell through permeabilization. Thus, pulsed electric fields can recruit two sources of calcium ions, i.e., extracellular and intracellular released from intracellular compartments, contributing to the enhanced anticancer effect.

## 5. Conclusions

We have demonstrated that nanosecond pulses increased cell membrane permeability independently of the drug resistance phenomenon. The permeability rate appeared to be dependent on the cell size and type. We could observe an enhanced antiproliferative effect in the presence of Ca^2+^ in electroporated cancer cells, which seems to be a safe anticancer drug in therapeutic protocols. The immunofluorescent studies revealed that the expression of P-gp was not downregulated. However, the most important observation was the decreased MDR1 activity after nanosecond electroporation, particularly calcium ions. According to our results, we presume the phenomenon of MDR mechanism disruption using electroporation in combination with calcium ions is promising. We suppose that PEF with calcium ions can reduce drug resistance by inhibiting drug efflux activity. Nevertheless, this statement and the effect of pulsed electric fields on drug resistance mechanisms require further studies in the future, including cells with varying lipid membrane composition and different contents of drug-resistant proteins.

## Figures and Tables

**Table 1:** The results of the titration for calcium and magnesium ions in experimental buffers.

Sample	Ca^2+^ & Mg^2+^ [mmol/L]	Ca^2+^ [mmol/L]	Mg^2+^ (= [Ca, Mg]−[Ca]) [mmol/L]
10 mM HEPES	0.017 ± 0.005	0.015 ± 0.006	0.00
10 mM HEPES + sucrose + 1 mmol/L Mg^2+^	0.96 ± 0.03	0.010 ± 0.009	0.95 ± 0.04
10 mM HEPES + sucrose + 1 mmol/L Mg^2+^ + 2 mmol/L Ca^2+^	2.64 ± 0.05	1.68 ± 0.02	0.96 ± 0.07
Milli-Q	0.00	0.00	0.00

## Data Availability

Data is contained within the article.

## References

[1] Omran Z., Scaife P., Stewart S., Rauch C. (2017). Physical and biological characteristics of multi drug resistance (MDR): An integral approach considering pH and drug resistance in cancer. Semin. Cancer Biol..

[2] Ye S., MacEachran D.P., Hamilton J.W., O’Toole G.A., Stanton B.A. (2008). Chemotoxicity of doxorubicin and surface expression of P-glycoprotein (MDR1) is regulated by the Pseudomonas aeruginosa toxin Cif. Am. J. Physiol. Physiol..

[3] Shen F., Chu S., Bence A.K., Bailey B., Xue X., Erickson P.A., Montrose M.H., Beck W.T., Erickson L.C. (2008). Quantitation of Doxorubicin Uptake, Efflux, and Modulation of Multidrug Resistance (MDR) in MDR Human Cancer Cells. J. Pharmacol. Exp. Ther..

[4] Ji X., Lu Y., Tian H., Meng X., Wei M., Cho W.C. (2019). Chemoresistance mechanisms of breast cancer and their countermeasures. Biomed. Pharmacother..

[5] Housman G., Byler S., Heerboth S., Lapinska K., Longacre M., Snyder N., Sarkar S. (2014). Drug resistance in cancer: An overview. Cancers.

[6] Briffa R., Langdon S.P., Grech G., Harrison D.J. (2017). Acquired and Intrinsic Resistance to Colorectal Cancer Treatment. Colorectal Cancer—Diagnosis, Screening and Management.

[7] Dartier J., Lemaitre E., Chourpa I., Goupille C., Servais S., Chevalier S., Mahéo K., Dumas J.F. (2017). ATP-dependent activity and mitochondrial localization of drug efflux pumps in doxorubicin-resistant breast cancer cells. Biochim. Biophys. Acta Gen. Subj..

[8] Pan S.T., Li Z.L., He Z.X., Qiu J.X., Zhou S.F. (2016). Molecular mechanisms for tumour resistance to chemotherapy. Clin. Exp. Pharmacol. Physiol..

[9] Fletcher J.I., Williams R.T., Henderson M.J., Norris M.D., Haber M. (2016). ABC transporters as mediators of drug resistance and contributors to cancer cell biology. Drug Resist. Updat..

[10] Robey R.W., Pluchino K.M., Hall M.D., Fojo A.T., Bates S.E., Gottesman M.M. (2018). Revisiting the role of ABC transporters in multidrug-resistant cancer. Nat. Rev. Cancer.

[11] Hu T., Li Z., Gao C.Y., Cho C.H. (2016). Mechanisms of drug resistance in colon cancer and its therapeutic strategies. World J. Gastroenterol..

[12] Mansoori B., Mohammadi A., Davudian S., Shirjang S., Baradaran B. (2017). The different mechanisms of cancer drug resistance: A brief review. Adv. Pharm. Bull..

[13] Qu N., Shi D., Shang M., Duan S., Guo L., Ning S., Li J. (2018). Breast cancer cell line phenotype affects sonoporation efficiency under optimal ultrasound microbubble conditions. Med. Sci. Monit..

[14] Kato S., Shirai Y., Sakamoto M., Mori S., Kodama T. (2019). Use of a Lymphatic Drug Delivery System and Sonoporation to Target Malignant Metastatic Breast Cancer Cells Proliferating in the Marginal Sinuses. Sci. Rep..

[15] Kranjc S., Kranjc M., Scancar J., Jelenc J., Sersa G., Miklavcic D. (2016). Electrochemotherapy by pulsed electromagnetic field treatment (PEMF) in mouse melanoma B16F10 in vivo. Radiol. Oncol..

[16] Rolong A., Davalos R.V., Rubinsky B. (2018). History of Electroporation. Irreversible Electroporation in Clinical Practice.

[17] Petrini M., Mattii L., Sabbatini A., Carulli G., Grassi B., Cadossi R., Ronca G., Conte A. (1990). Multidrug resistance and electromagnetic fields. Electromagn. Biol. Med..

[18] Pickering S.A.W., Bayston R., Scammell B.E. (2003). Electromagnetic augmentation of antibiotic efficacy in infection of orthopaedic implants. J. Bone Jt. Surg. Ser. B.

[19] Segatore B., Setacci D., Bennato F., Cardigno R., Amicosante G., Iorio R. (2012). Evaluations of the effects of extremely low-frequency electromagnetic fields on growth and antibiotic susceptibility of escherichia coli and pseudomonas aeruginosa. Int. J. Microbiol..

[20] Saczko J., Pilat J., Choromanska A., Rembialkowska N., Bar J., Kaminska I., Zalewski J., Kulbacka J. (2016). The effectiveness of chemotherapy and electrochemotherapy on ovarian cell lines in vitro. Neoplasma.

[21] Campana L.G., Edhemovic I., Soden D., Perrone A.M., Scarpa M., Campanacci L., Cemazar M., Valpione S., Miklavčič D., Mocellin S. (2019). Electrochemotherapy-Emerging applications technical advances, new indications, combined approaches, and multi-institutional collaboration. Eur. J. Surg. Oncol..

[22] Batista Napotnik T., Reberšek M., Vernier P.T., Mali B., Miklavčič D. (2016). Effects of high voltage nanosecond electric pulses on eucaryotic cells (in vitro): A systematic review. Bioelectrochemistry.

[23] Gianulis E.C., Labib C., Saulis G., Novickij V., Pakhomova O.N., Pakhomov A.G. (2016). Selective susceptibility to nanosecond pulsed electric field (nsPEF) across different human cell types. Cell. Mol. Life Sci..

[24] Romeo S., Sannino A., Scarfì M.R., Vernier P.T., Cadossi R., Gehl J., Zeni O. (2018). ESOPE-Equivalent Pulsing Protocols for Calcium Electroporation: An In Vitro Optimization Study on 2 Cancer Cell Models. Technol. Cancer Res. Treat..

[25] Frandsen S.K., Gissel H., Hojman P., Eriksen J., Gehl J. (2014). Calcium electroporation in three cell lines: A comparison of bleomycin and calcium, calcium compounds, and pulsing conditions. Biochim. Biophys. Acta Gen. Subj..

[26] Falk H., Matthiessen L.W., Wooler G., Gehl J. (2018). Calcium electroporation for treatment of cutaneous metastases; a randomized double-blinded phase II study, comparing the effect of calcium electroporation with electrochemotherapy. Acta Oncol..

[27] Zhao J., Wen X., Tian L., Li T., Xu C., Wen X., Melancon M.P., Gupta S., Shen B., Peng W. (2019). Irreversible electroporation reverses resistance to immune checkpoint blockade in pancreatic cancer. Nat. Commun..

[28] Cemazar M., Miklavcic D., Mir L.M., Belehradek J., Bonnay M., Fourcault D., Sersa G. (2001). Electrochemotherapy of tumours resistant to cisplatin: A study in a murine tumour model. Eur. J. Cancer.

[29] Marty M., Sersa G., Garbay J.R., Gehl J., Collins C.G., Snoj M., Billard V., Geertsen P.F., Larkin J.O., Miklavcic D. (2006). Electrochemotherapy—An easy, highly effective and safe treatment of cutaneous and subcutaneous metastases: Results of ESOPE (European Standard Operating Procedures of Electrochemotherapy) study. Eur. J. Cancer Suppl..

[30] Grandi M., Geroni C., Giuliani F.C. (1986). Isolation and characterization of a human colon adenocarcinoma cell line resistant to doxorubicin. Br. J. Cancer.

[31] Jeffrey G., Bassett J., Denney R. (1989). Determination of Calcium and Magnesium. Vogel’s Textbook of Quantitative Chemical Analysis.

[32] Jeffery G., Bassett J., Denney R. (1989). Complexation Titrations. Vogel’s Textbook of Quantitative Chemical Analysis.

[33] Harris D.C., Lucy C.A. (2015). EDTA Titrations. Daniel C. Harris: Quantitative Chemical Analysis.

[34] Novickij V., Rembialkowska N., Staigvila G., Kulbacka J. (2020). Effects of extracellular medium conductivity on cell response in the context of sub-microsecond range calcium electroporation. Sci. Rep..

[35] Schindelin J., Arganda-Carreras I., Frise E., Kaynig V., Longair M., Pietzsch T., Preibisch S., Rueden C., Saalfeld S., Schmid B. (2012). Fiji: An open-source platform for biological-image analysis. Nat. Methods.

[36] Forster S., Thumser A.E., Hood S.R., Plant N. (2012). Characterization of rhodamine-123 as a tracer dye for use in in vitro drug transport assays. PLoS ONE.

[37] Moreira H., Szyjka A., Gasiorowski K. (2018). Chemopreventive activity of celastrol in drug-resistant human colon carcinoma cell cultures. Oncotarget.

[38] Adkins C.E., Mittapalli R.K., Manda V.K., Nounou M.I., Mohammad A.S., Terrell T.B., Bohn K.A., Yasemin C., Grothe T.R., Lockman J.A. (2013). P-glycoprotein mediated efflux limits substrate and drug uptake in a preclinical brain metastases of breast cancer model. Front. Pharmacol..

[39] Wang Y., Hao D.C., Stein W.D., Yang L. (2006). A kinetic study of Rhodamine123 pumping by P-glycoprotein. Biochim. Biophys. Acta Biomembr..

[40] Twentyman P.R., Rhodes T., Rayner S. (1994). A comparison of rhodamine 123 accumulation and efflux in cells with P-glycoprotein-mediated and MRP-associated multidrug resistance phenotypes. Eur. J. Cancer.

[41] Frandsen S.K., Vissing M., Gehl J. (2020). A comprehensive review of calcium electroporation—A novel cancer treatment modality. Cancers.

[42] Zielichowska A., Daczewska M., Saczko J., Michel O., Kulbacka J. (2016). Applications of calcium electroporation to effective apoptosis induction in fibrosarcoma cells and stimulation of normal muscle cells. Bioelectrochemistry.

[43] Lastauskiene E., Novickij V., Zinkevičiene A., Girkontaite I., Paškevičius A., Švediene J., Markovskaja S., Novickij J. (2019). Application of pulsed electric fields for the elimination of highly drug-resistant Candida grown under modelled microgravity conditions. Int. J. Astrobiol..

[44] Wang J., Guo J., Wu S., Feng H., Sun S., Pan J., Zhang J., Beebe S.J. (2012). Synergistic Effects of Nanosecond Pulsed Electric Fields Combined with Low Concentration of Gemcitabine on Human Oral Squamous Cell Carcinoma In Vitro. PLoS ONE.

[45] Kunjachan S., Rychlik B., Storm G., Kiessling F., Lammers T. (2013). Multidrug resistance: Physiological principles and nanomedical solutions. Adv. Drug Deliv. Rev..

[46] Ozben T. (2006). Mechanisms and strategies to overcome multiple drug resistance in cancer. FEBS Lett..

[47] Choromańska A., Chwiłkowska A., Kulbacka J., Baczyńska D., Rembiałkowska N., Szewczyk A., Michel O., Gajewska-Naryniecka A., Przystupski D., Saczko J. (2021). Modifications of Plasma Membrane Organization in Cancer Cells for Targeted Therapy. Molecules.

[48] Agarwal A., Zudans I., Weber E.A., Olofsson J., Orwar O., Weber S.G. (2007). Effect of cell size and shape on single-cell electroporation. Anal. Chem..

[49] Henslee B.E., Morss A., Hu X., Lafyatis G.P., Lee L.J. (2011). Electroporation dependence on cell size: Optical tweezers study. Anal. Chem..

[50] Kulbacka J., Daczewska M., Dubińska-Magiera M., Choromańska A., Rembiałkowska N., Surowiak P., Kulbacki M., Kotulska M., Saczko J. (2014). Doxorubicin delivery enhanced by electroporation to gastrointestinal adenocarcinoma cells with P-gp overexpression. Bioelectrochemistry.

[51] Weżgowiec J., Kulbacka J., Saczko J., Rossowska J., Chodaczek G., Kotulska M. (2018). Biological effects in photodynamic treatment combined with electropermeabilization in wild and drug resistant breast cancer cells. Bioelectrochemistry.

[52] Hoejholt K.L., Mužić T., Jensen S.D., Dalgaard L.T., Bilgin M., Nylandsted J., Heimburg T., Frandsen S.K., Gehl J. (2019). Calcium electroporation and electrochemotherapy for cancer treatment: Importance of cell membrane composition investigated by lipidomics, calorimetry and in vitro efficacy. Sci. Rep..

[53] Levine Z.A., Vernier P.T. (2012). Calcium and phosphatidylserine inhibit lipid electropore formation and reduce pore lifetime. J. Membr. Biol..

[54] Boettcher J.M., Davis-Harrison R.L., Clay M.C., Nieuwkoop A.J., Ohkubo Y.Z., Tajkhorshid E., Morrissey J.H., Rienstra C.M. (2011). Atomic view of calcium-induced clustering of phosphatidylserine in mixed lipid bilayers. Biochemistry.

[55] Pakhomova O.N., Gregory B., Semenov I., Pakhomov A.G. (2014). Calcium-mediated pore expansion and cell death following nanoelectroporation. Biochim. Biophys. Acta Biomembr..

[56] Cemazar I.M., Sersa G., Ycld D.M. (1998). Electrochemotherapy with Cisplatin in the Treatment of Tumor Cells Resistant to Cisplatin. Anticancer Res..

[57] Condello M., D’Avack G., Spugnini E.P., Meschini S. (2019). View of Electroporation: New strategy to improve the drug uptake and overcome the tumour resistance. Microscopie.

[58] Semenov I., Xiao S., Pakhomova O.N., Pakhomov A.G. (2013). Recruitment of the intracellular Ca2+ by ultrashort electric stimuli: The impact of pulse duration. Cell Calcium.

[59] Napotnik T.B., Wu Y.H., Gundersen M.A., Miklavčič D., Vernier P.T. (2012). Nanosecond electric pulses cause mitochondrial membrane permeabilization in Jurkat cells. Bioelectromagnetics.

[60] Zharkova L.P., Romanchenko I.V., Buldakov M.A., Priputnev P.V., Bolshakov M.A., Rostov V.V. (2018). Mitochondrial Membrane Permeability after Nanosecond Electromagnetic Pulsed Exposure. Proceedings of the 2018 20th International Symposium on High-Current Electronics, ISHCE 2018.

[61] Vernier P.T., Sun Y., Gundersen M.A. (2006). Nanoelectropulse-driven membrane perturbation and small molecule permeabilization. BMC Cell Biol..

